# Big data analytics on social networks for real-time depression detection

**DOI:** 10.1186/s40537-022-00622-2

**Published:** 2022-05-20

**Authors:** Jitimon Angskun, Suda Tipprasert, Thara Angskun

**Affiliations:** 1 School of Information Technology, Suranaree University of Technology, Nakhon Ratchasima, Thailand; 2 DIGITECH, Suranaree University of Technology, Nakhon Ratchasima, Thailand

**Keywords:** Big data analytics, Depression detection, Social networks

## Abstract

During the coronavirus pandemic, the number of depression cases has dramatically increased. Several depression sufferers disclose their actual feeling via social media. Thus, big data analytics on social networks for real-time depression detection is proposed. This research work detected the depression by analyzing both demographic characteristics and opinions of Twitter users during a two-month period after having answered the Patient Health Questionnaire-9 used as an outcome measure. Machine learning techniques were applied as the detection model construction. There are five machine learning techniques explored in this research which are Support Vector Machine, Decision Tree, Naïve Bayes, Random Forest, and Deep Learning. The experimental results revealed that the Random Forest technique achieved higher accuracy than other techniques to detect the depression. This research contributes to the literature by introducing a novel model based on analyzing demographic characteristics and text sentiment of Twitter users. The model can capture depressive moods of depression sufferers. Thus, this work is a step towards reducing depression-induced suicide rates.

## Introduction

Depression means “a state of mind that expresses mood disorders such as depressed, unhappy, bored, loss of appetite, lack of concentration, anxiety, etc.”. Depression may adversely affect the quality of life and it can eventually lead to suicide [1]. Depression can cause of significant health deterioration and affect people all over the world. The World Health Organization estimated that there were approximately 280 million people in 2021 who suffered from depression. In low-income and middle-income countries, 80% of them were untreated [2]. In the United States, the depression cases have tripled during the coronavirus pandemic [3]. While in Thailand, the number of depression cases continues to increase and 60% of suicides are caused by depression. Therefore, the prevention, treatment, and promotion of mental health are very important [4].

As mentioned above, all people have a risk of depression and most of them do not receive treatment because they do not know that they are depressed. Moreover, friends and family members may do not know either. People who are at risk of depression often express themselves through social networks. Social networking is a form of communication that does not rely on eye contact and facial expression, but it can be expressed through commenting on messages or pictures [5]. Hence, social media data can detect depression of individuals based on their posted negative opinions [6].

Nevertheless, the nature of social media data is big data in terms of large volumes and high velocity of data [7]. Big data can be processed in two approaches which are big data batch processing and big data stream processing [8]. In this research, the big data batch processing is suitable for creating a depression detection model, while the big data stream processing is necessary for real-time depression detection. The reason being not only the large volume of data need to be computed but also that data must be rapidly processed in real-time to reduce depression-induced suicide rates.

Machine learning techniques [9] were applied as the detection model construction. There are five machine learning techniques explored in this research which are Support Vector Machine, Decision Tree, Random Forest, Naïve Bayes, and Deep Learning. Support Vector Machine tries to choose the optimal decision boundary (i.e., hyperplane) by maximizing the margin distance between classes using Hinge Loss function as defined by the equation $l(y) = \max (0, 1 + \max _{y \ne t} w_{y}x - w_{t}x )$ where *t* is the target label, $w_{t}$ and $w_{y}$ are the model parameters. Decision tree divides the dataset based on the attribute that divides the dataset most effectively. The attribute which provides maximum Information Gain is selected for splitting. Information Gain is the entropy of the parent node minus the sum of entropy of the child nodes, where entropy is defined as $Entropy = \sum _{i=1}^{c} -P_{i} * log_{2}P_{i}$. Random forest is composed of several decision trees, which operate as an ensemble. Each decision tree predicts a class outcome. The class outcome with the most number of votes is the prediction of random forest. Thus, the decision trees should be least correlated with each other, i.e., each decision tree predicts using different features. Naïve Bayes classifies data based on Bayes’s theorem. If $C_{k}$ represents possible classes, and vector *x* represents features, the conditional probability can be defined as $P(C_{k}|x) = \frac{P(x|C_{k}) P(C_{k})}{P(x)}$, where $P(C_{k}|x)$ is posterior, $P(x|C_{k})$ is likelihood, $P(C_{k})$ is prior, and *P*(*x*) is evidence. Deep Learning is based on artificial neural networks, inspired by information processing and distributed communication nodes in biological brain. It uses multiple hidden layers to extract features from inputs. An output of a neural is the activation function *f* of a weighted sum of the neuron’s input i.e., output is $f (b + \sum _{i=1}^{n} x_{i}w_{i})$, where $w_{i}$ is a corresponding weight of input $x_{i}$, and *b* is bias.

## Literature review

There are several existing research works focusing on depression detection using social network. These works can be categorized into three groups based on their objective. The first group (e.g., [10–14]) aims to analyze feelings from social network data. Barhan and Shakhomirov [10] developed a model to classify feelings from Twitter data using emoticons and sentiments based on *N*-gram, a technique used to calculate the probabilities of words based on the preceding *n*-1 words from the target word. The results showed that Support Vector Machine performed better than Naïve Bayes. It has 81% accuracy and 74% recall. The result was consistent with that of Hutto and Gilbert [11]’s work, which presented performance comparison of sentiment analysis algorithms for analyzing social network messages. Four thousand Twitter messages were analyzed using Support Vector Machine, Naïve Bayes, and maximum entropy algorithms. The result indicated that Support Vector Machine achieved the highest accuracy at 91%. Vateekul and Koomsubha [12] introduced a new approach to apply a deep learning technique to classify sentiments of Thai Twitter data. The results revealed that deep learning technique achieved higher accuracy than traditional techniques including Naïve Bayes and Support Vector Machine. Islam et al. [13] analyzed the user feelings from social networks to examine their moods and attitudes when communicating through these online tools. Machine learning techniques were investigated for depression detection. The results showed that decision tree was highly accurate for identifying mental health problems. Sood et al. [14] presented a method for analyzing sentiment to identify depression. They used R Studio to extract data from Twitter. A sentiment score was derived from Twitter messages to indicate individual happiness. The results showed that sentiment analysis from Twitter could help to screen depression among general public.

The second group (e.g., [15–17]) intends to detect depression from social network data using various approaches. Park et al. [15] proposed a framework for analyzing sentiments from Twitter and searching for features that identified depression. The experimental results with 69 participants found that using negative messages, messages expressing depression, negative Twitter emoticon (a.k.a. emotion icon) was significantly associated with the occurrence of depression symptoms. Jiang et al. [16] examined the acoustic correlations of a sample of 170 participants (85 of them were depressed patients). They examined three different types of speech (interviews, picture description, and reading) and three different emotions (positive, neutral, and negative) to detect depression. The study found that picture description provided better classification results than others. The results also indicated that speech and emotions had potential for detecting depression. Orabi et al. [17] pinpointed the most effective deep learning architecture for natural language processing task. The designated architecture was used to detect depression from unstructured data of Twitter users.

The third group (e.g., [18–21]) concentrates on constructing a depression detection model. Hu et al. [18] created classification and regression models using behavioral and linguistic data from 10,102 social network users. The results revealed that data from social networks could be used to predict the depression in advance up to two months. Aldarwish and Ahmad [19] conducted their research on predicting depression levels using comment messages from social networks. They used data mining techniques to predict levels of mental health and depression from comment messages. The classification model was created using Support Vector Machine and Naïve Bayes techniques. The accuracy value was 63.30%. Reece et al. [20] created a depression prediction model based on images from Instagram using the Bayesian Logistic Regression algorithm. A sample of 166 participants and 43,950 images were gathered for color analysis and face detection. The experimental results showed that the model achieved better efficiency than conventional diagnostics with an overall efficiency of 61%. Sun et al. [21] proposed a hybrid model, which was a combination of the Convolutional Neural Network Long Short-Term Memory and the Markov chain Monte Carlo methods. The model was used to identify user feelings, mood changes, and mood disorders. The experimental results revealed that the model could detect irregular conversion and emotional disorders.

The objective of this work is all the three groups. A comparison of research related to depression detection using social network is shown in Table 1. In terms of the analytical features, the existing research focused on only some information of Twitter users such as messages (e.g., tweets, retweets, hashtags), messages expressing depression (called sentiments), emoticons, images, Twitter behaviors, or user profiles (called demographic characteristics). For measuring success of depression detection, the existing research applied self-reported survey responses, self-declared mental health status, forum membership, or annotated posts as the indicator. The self-reported surveys are second only to clinical interviews for depression detection accuracy [22]. In terms of model development techniques, it was found that most research created a model using only single technique.

Therefore, this research applies the demographic characteristics of Twitter users, and their opinions or tweets during a two-month period after answering their self-reported survey (the Patient Health Questionnaire-9: PHQ-9) to develop a depression detection model. The demographic characteristics and tweets are used as features or inputs for the model construction, while the self-reported survey responses are used as the outcome measure. Additionally, the proposed model applies a variety of machine learning techniques including Support Vector Machine, Naïve Bayes, Decision Tree, Deep Learning, and Random Forest techniques. The most suitable features and techniques will be chosen for the model construction. After the model construction, the model is used in a process of real-time depression detection.

**Table 1:** A comparison of research related to depression detection using social network

Depression detection	Related work												This work
	[10]	[11]	[12]	[13]	[14]	[15]	[16]	[17]	[18]	[19]	[20]	[21]	
Data source													
Facebook				✓						✓			
Instagram											✓		
Sina Weibo									✓				
Twitter	✓	✓	✓		✓	✓	✓	✓				✓	✓
Input feature													
Message	✓	✓	✓	✓	✓	✓	✓	✓	✓	✓		✓	✓
Sentiment	✓	✓	✓	✓	✓	✓	✓			✓		✓	✓
Emoticon	✓					✓			✓				
Image											✓		
Behavior									✓				✓
Profile						✓			✓				✓
Methodology													
Decision Tree				✓	✓								✓
Deep Learning			✓					✓				✓	✓
K-Nearest Neighbors				✓									
Maximum Entropy		✓											
Naïve Bayes	✓	✓	✓							✓			✓
Random Forest											✓		✓
Regression						✓			✓				
Support Vector Machine	✓	✓	✓	✓						✓			✓
Result													
Positive/negative	✓	✓	✓	✓	✓	✓	✓	✓	✓	✓	✓	✓	✓
Depression level													✓

## Real-time depression detection methodology

**Fig. 1:**
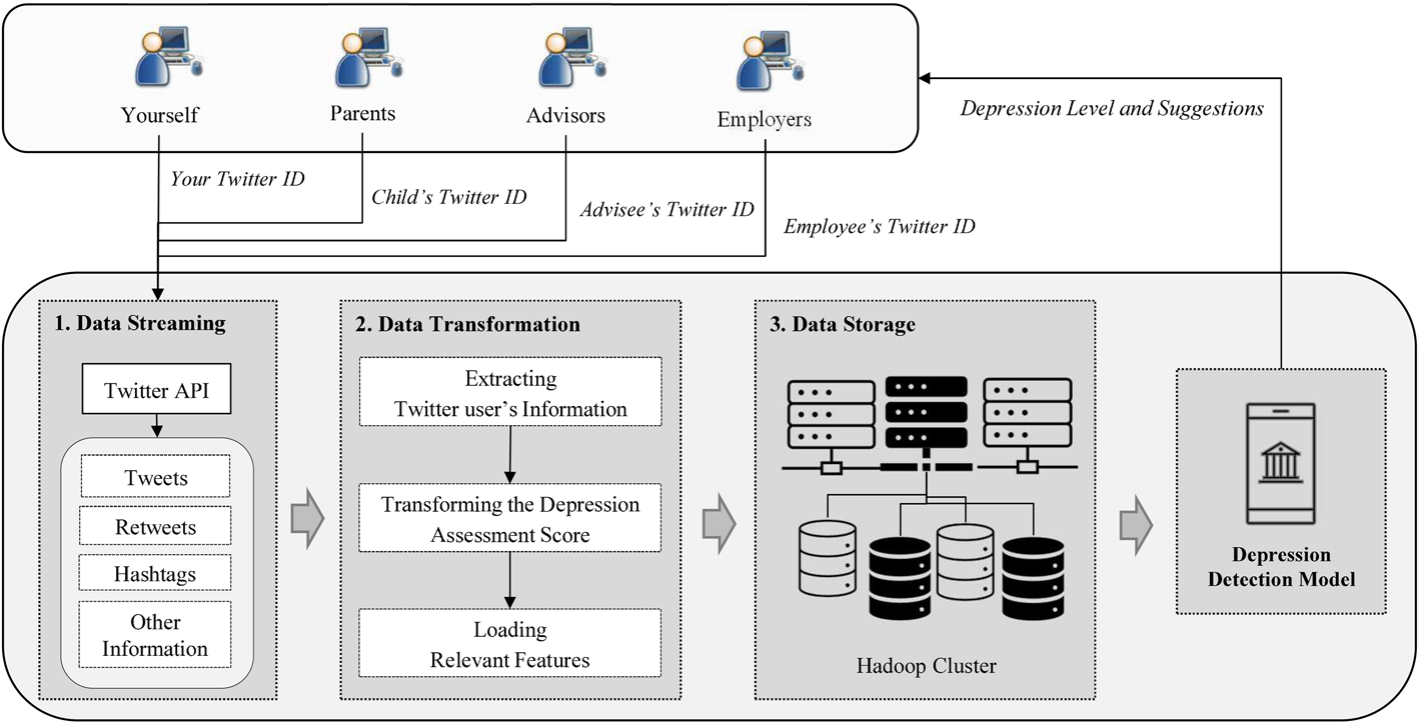
The process of real-time depression detection

Real-time depression detection is a process where gathers streaming data from Twitter Application Programming Interface (API) by extracting, transforming and loading data into data storage in a Hadoop cluster. These data will be used as an unseen input data of the depression detection model. The model is used as a classifier to identify a depression level of a person. There are four modes of users in the system called yourself, parent, advisor, and employer. In the yourself mode, users can use their own Twitter ID as an input for the system to detect their own depression level. In the parent mode, parents can track a status of their child by providing child’s twitter ID to the system. In the advisor mode, advisors can track a depression level of their advisee by supplying Twitter ID of advisee into the system. In the employer mode, employers can also track a status of their employee by providing employee’s twitter ID to the system. Please note that advisors and employers must obtain a permission from their advisee and employee, respectively, in order to track their depression level. The process of real-time depression detection is shown in Fig. 1.

## Depression detection model

**Fig. 2:**
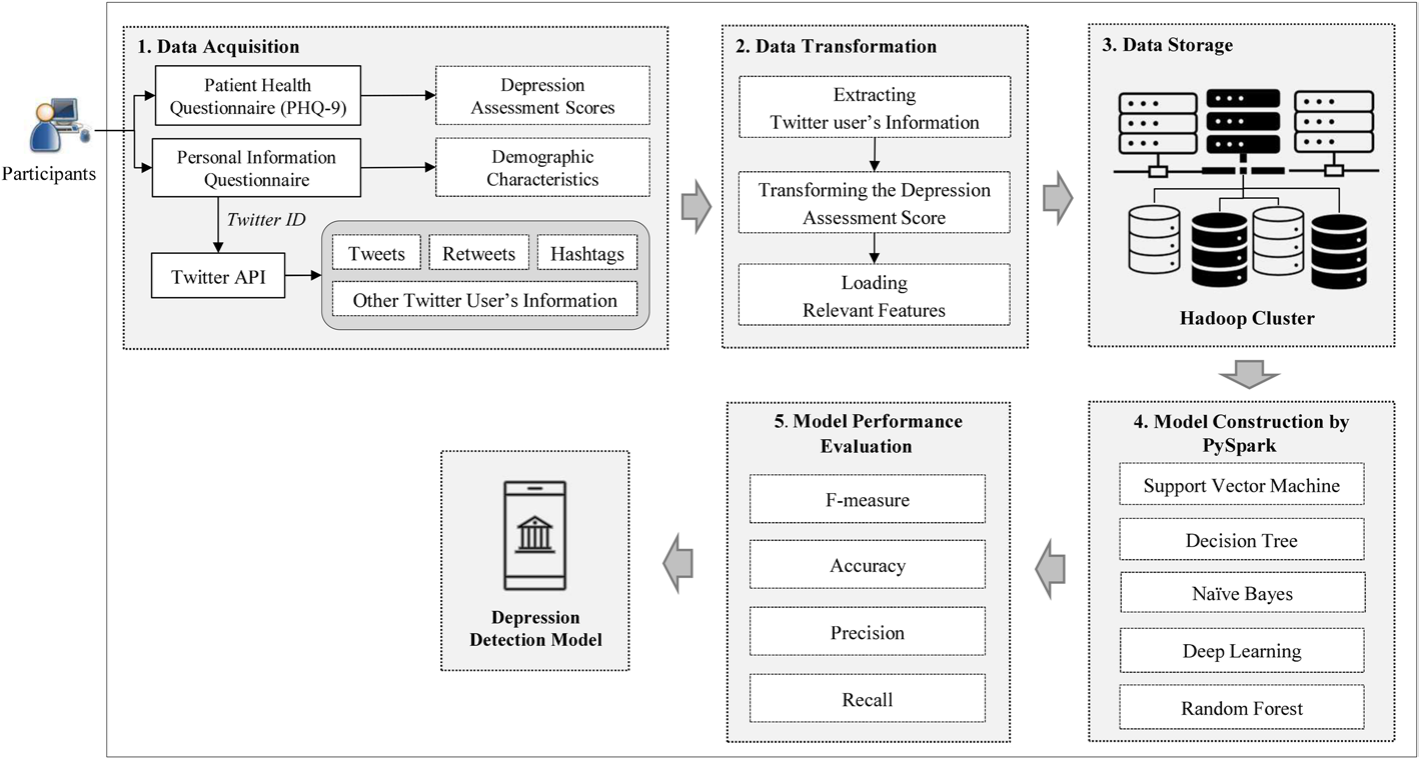
The framework of a depression detection model

This research aims to develop a depression detection model using demographic characteristics and sentiment analysis from tweets. The research methodology consists of 5 processes as shown in Fig. 2 which are data acquisition, data transformation, data storage, model construction, and model performance evaluation. The details of each process are described as follows.

### Data acquisition

The data acquisition for model construction in this research is obtained from three sources which are the PHQ-9, a personal information questionnaire, and Twitter API.

Firstly, the data derived from the PHQ-9 are survey response date and a depression assessment score of each participant.

Secondly, the data collected from the personal information questionnaire comprise Twitter ID and demographic characteristics such as gender, age, weight, education, congenital disease, career, income, number of family members, self-couple status, and parent’s marital status.

Finally, Twitter API is used to collect Twitter user’s information in real time from the Twitter page. The Twitter ID collected from the first source is used to access the Twitter user’s information. The information was gathered 2 months prior to the survey response date. There are 222 tweets, 1522 retweets and 16 hashtags, collected from 192 Twitter users, 50 of whom are moderately depressed, 74 are slightly depressed, and 68 show no sign of being depressed.

### Data transformation

The data processing for model construction using machine learning consists of three steps which are detailed as follows.

#### Extracting Twitter user’s information

**Fig. 3:**
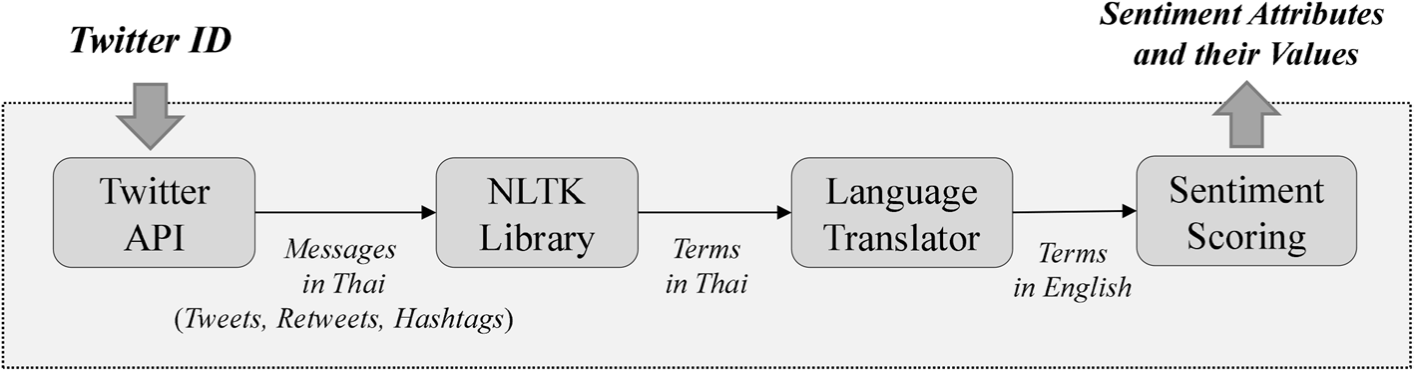
The process of feature extraction

The information obtained from Twitter will be processed to find sentiment attributes and their values. The process of extraction is illustrated in Fig. 3 and can be explained as follows. Firstly, the Twitter ID is used to access the Twitter user’s information via Twitter API. The extracted information consists of tweets, retweets, hashtags, the number of friends, the number of followers, and periods of tweets. Secondly, terms in the tweets, retweets, and hashtags are extracted using an NLTK library [23]. Thirdly, the extracted terms in Thai are translated to English using Google translation API. Finally, the sentiment numerical scores of each term are derived from opinion lexicons in the WordNet database [24], where each term has three sentiment numerical scores: *Pos*(*s*), *Neg*(*s*) and *Obj*(*s*), according to Eq. (1).

1 $$Pos(s) + Neg(s) + Obj(s) = 1 $$

The extracted sentiment attributes in this research comprise the number of positive and negative tweets, retweets and hashtags, the number of tweets, retweets and hashtags expressing depression, and sentiment score of all tweets.

The sentiment score of all tweets $\Theta$ is derived from sentiment scores of each tweet *i* as shown in Eq. (2).

2 $$ \Theta = \frac{\sum _{i=1}^{n} Tweet(s)_{i}}{n}$$

The sentiment score of each tweet $Tweet(s)_{i}$ is calculated from an average of positive and negative scores of all terms in the tweet as shown in Eq. (3).

3 $$ Tweet(s) = \frac{\sum _{j=1}^{m} Pos(s)_{j}}{m} - \frac{\sum _{j=1}^{m} Neg(s)_{j}}{m} $$

The number of positive tweets $Tweet_{pos}$ is the total number of tweets that have $Tweet(s) > 0$. On the other hand, the number of negative tweets $Tweet_{neg}$ is the total number of tweets that have $Tweet(s) \ge 0$. The number of tweets expressing depression $Tweet_{depress}$ is the total number of tweets that have an occurrence of at least one of depressive term. There are 78 depressive words collected from the PHQ-9 questionnaire and stored in a depression term corpus [25].

The number of positive and negative retweets and hashtags, and the number of retweets and hashtags expressing depression are derived in the same way as those equation of tweets.

#### Transforming the depression assessment score

The scores obtained from the PHQ-9 are converted from numeric to nominal. The scores are converted in to four classes as follows: 5-8 points are converted to Level 0 or no depression, 9-14 points are converted to Level 1 or slight depression, and more than 15 points are converted to Level 2 or moderate depression.

#### Loading relevant features

Before loading features into data storage, attributes obtained from the previous step must be relevant features. Feature selection is the process of selecting a subset of relevant features for model construction. The relevant features as shown in Table 2 include 10 demographic characteristic attributes ($x_{1},\ldots,x_{10}$), 10 sentiment attributes ($x_{11},\ldots,x_{20}$), 4 Twitter user’s information attributes ($x_{21},\ldots,x_{24}$), and depression evaluation result attribute (*y*). Moreover, the table contains additional details and data types of each feature. Irrelevant data such as Twitter ID and the survey response date are eliminated to prevent unnecessary computation in the machine learning technique. These relevant features will be loaded into the next process.

**Table 2:** Data characteristics of relevant features

Attribute ID	Attribute name	Data type	Description
$x_{1}$	Gender	Nominal	0: female, 1: male, 2: others
$x_{2}$	Age	Nominal	1: < 20, 2: 20–29, 3: 30–39, 4: 40–49, 5: 50–59, 6: ≥ 60
$x_{3}$	Weight	Nominal	1: < 40, 2: 40–59, 3: 60–79, 4: 80–99, 5: ≥ 100
$x_{4}$	Education	Nominal	1: high school diploma or less, 2: bachelor’s degree, 3: graduate degree
$x_{5}$	CongenitalDisease	Nominal	1: no, 2: yes
$x_{6}$	Career	Nominal	1: government official, 2: state enterprise employee, 3: businessman, 4: student, 5: homemaker, 6: not currently employed
$x_{7}$	Income	Nominal	1: sufficient, 2: insufficient
$x_{8}$	FamilyMember	Numeric	Number of family members
$x_{9}$	Self-CoupleStatus	Nominal	1: no couple, 2: stay together with couple, 3: separated with couple
$x_{10}$	ParentMaritalStatus	Nominal	1: stay together with couple, 2: separated with couple, 3: couple died
$x_{11}$	PositiveTweets	Numeric	Number of positive tweets
$x_{12}$	NegativeTweets	Numeric	Number of negative tweets
$x_{13}$	DepressionTweets	Numeric	Number of tweets expressing depression
$x_{14}$	PositiveRetweets	Numeric	Number of positive retweets
$x_{15}$	NegativeRetweets	Numeric	Number of negative retweets
$x_{16}$	DepressionRetweets	Numeric	Number of retweets expressing depression
$x_{17}$	PositiveHashtags	Numeric	Number of positive hashtags
$x_{18}$	NegativeHashtags	Numeric	Number of negative hashtags
$x_{19}$	DepressionHashtags	Numeric	Number of hashtags expressing depression
$x_{20}$	SentimentScore	Numeric	Sentiment score of tweets
$x_{21}$	Friends	Numeric	Number of friends
$x_{22}$	Followers	Numeric	Number of followers
$x_{23}$	TweetPeriod1	Numeric	Number of tweets between 6 am and midnight
$x_{24}$	TweetPeriod2	Numeric	Number of tweets between midnight and 6 am
*y*	DepressionLevel	Nominal	Level 0: no depression, level 1: slight depression, level 2: depression

### Data storage

**Fig. 4:**
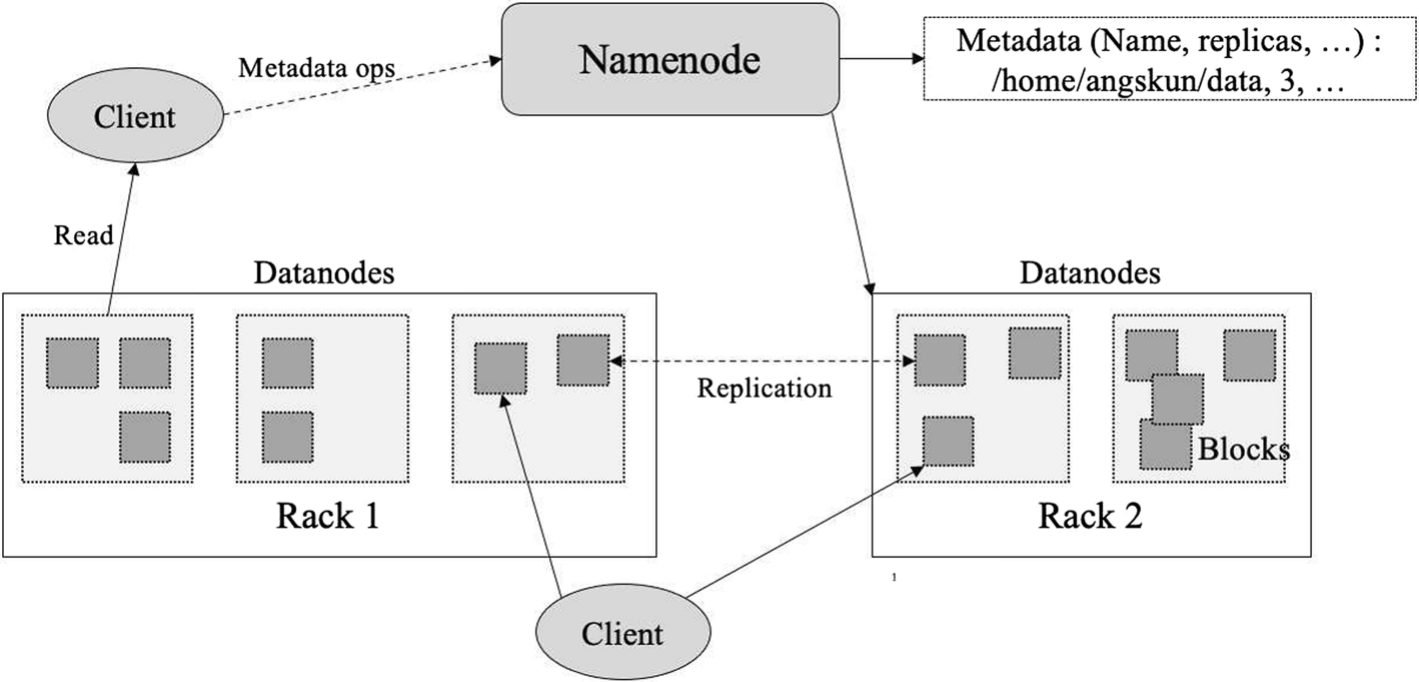
HDFS architecture

Data from the previous process are loaded into a Hadoop cluster, a specialized computer cluster designed for storing and analyzing a large amount of data. This research employed a Hadoop’s open source software suite called Cloudera running on commodity computers. Data gathered from Twitter are kept in Hadoop Distributed File System (HDFS). The HDFS consists of two principal components called name node and data node. Name node is served as metadata, where data nodes store the actual data block. Files are kept in 128-MB of data block, where each block is replicated on three different data nodes as illustrated in Fig. 4. Hadoop cluster is served as data lake for storing unstructured data from the Twitter.

### Model construction

Machine learning is used to create the automated depression detection model. The type of machine learning applied for the model construction is a classification technique. A set of data called the training set of the model uses features or input variables ($x_{1},\ldots,x_{24}$), and the target or output variable (*y*), all of which are listed in Table 2. More specifically, this research employs supervised machine learning techniques for model creation, including Support Vector Machine, Naïve Bayes, Decision Tree, Random Forest and Deep Learning techniques. Model construction is implemented with Spark MLlib, Spark’s machine learning library using Python interface. An advantage of Spark MLlib is that it can load a huge data set directly from the HDFS. This enables a large volume of tweets to process using machine learning.

### Model performance evaluation

The last process is to evaluate the performance of depression detection model to find the most appropriate machine learning technique for detecting the depression. The Support Vector Machine technique is used as a baseline of accuracy comparison since the majority of previous research work has relied on it [10–13, 19]. Comparing the results of different techniques is measured by standard measure scores which are f-measure, accuracy, precision, and recall [26].

## Experimental evaluation

### Testing environment

The assessment uses the same test set as the training set for the model construction with 192 Twitter users consisting of 3 classes (i.e., 50 moderately depressed people, 74 slightly depressed people, and 68 regular people). The tenfold cross validation approach is applied to evaluate the designed model. The Twitter user’s information consists of 222 tweets, 1,522 retweets and 16 hashtags. In addition, the test set also includes demographic characteristics.

The depression detection model has been assessed in three aspects. The first focuses on the selection of attributes (called features) that contributes to depression. This assessment compares the use of demographic characteristic features, features extracted from Twitter user’s information, and mixed features in order to construct the depression detection model.

The second aspect aims to reduce the number of features and the computational cost of modeling in order to improve the performance of the model. The process of reducing the number of features is called feature selection, which is applied to find the relevant features and the effective feature selection method for model construction.

The third aspect looks at the performance comparison of several machine learning techniques which are Support Vector Machine using the Radial Basis Function (RBF) kernel, Decision Tree using C4.5 algorithm, Naïve Bayes, Random Forest using 100 trees in the forest, and Deep Learning using deep feedforward networks with 100 hidden layers and ReLU activation function. Those techniques are compared by using scoring measures including F-measure, accuracy, precision, and recall [26] as shown in Eq. (4)–(7):

4 $$ Accuracy= \frac{TP + TN}{(TP + TN + FP + FN)} $$

5 $$Precision= \frac{TP}{(TP + FP)} $$

6 $$Recall=  \frac{TP}{(TP + FN)} $$

7 $${F-measure}= 2 \times \frac{Precision \times Recall}{Precision + Recall}$$

For depression level 0 (no depression), the *TP* (True Positive) means that persons with no sign of being depressed are correctly predicted as no depression. The *FP* (False Positive) means that persons who are slightly or moderately depressed are incorrectly predicted as no depression (actual depression level is 1 or 2). The *FN* (False Negative) means that persons with no sign of being depressed are incorrectly predicted as depression level 1 or 2 (actual depression level is 0). The *TN* (True Negative) means that persons who are slightly or moderately depressed are correctly predicted as depression level 1 or 2. The *TP*, *FP*, *FN*, *TN* of depression level 1 and 2 are derived in the same way as those of depression level 0.

The F-measure is used together with the accuracy since data is imbalanced. F-measure is a measure of a test’s accuracy. It is the harmonic mean of the precision and recall. The F-measure can be optimistic on severely imbalanced classification in problems with few samples of the minority class.

### Experimental results and discussion

**Fig. 5:**
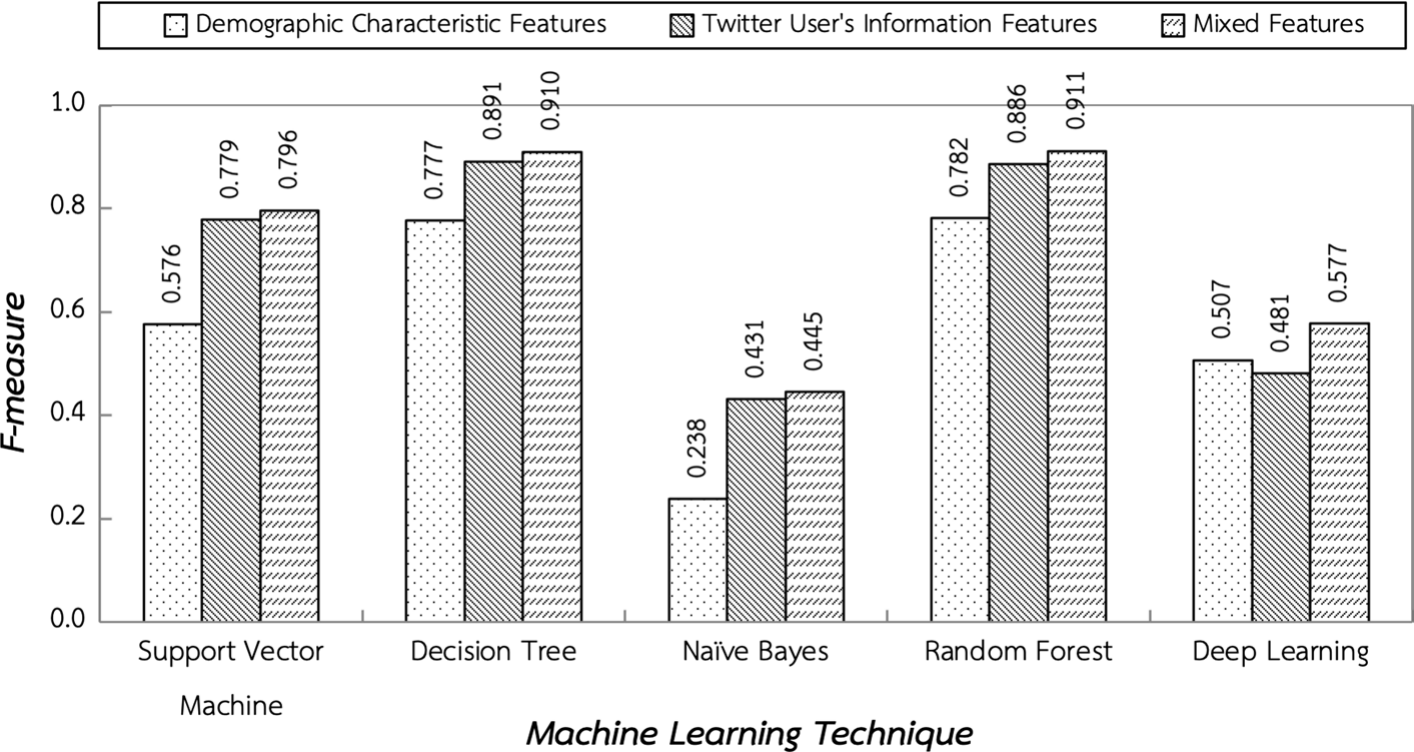
The effect of different features on performance of a variety of machine learning techniques

This section presents and discusses the experimental results based on the testing environment in the previous section. The first experiment aims to determine the effect of different features on performance of a variety of machine learning techniques including Support Vector Machine, Decision Tree, Naïve Bayes, Random Forest, and Deep Learning techniques. The results shown in Fig. 5 revealed that mixed features, a combination of demographic characteristics and Twitter user’s information features, achieved the highest average f-measure for all machine learning techniques at 0.728. Thus, this research employed 24 features from mixed features to construct the depression detection model.

In order to improve depression detection performance, feature selection was applied to reduce number of features or discover relevant features.

**Fig. 6:**
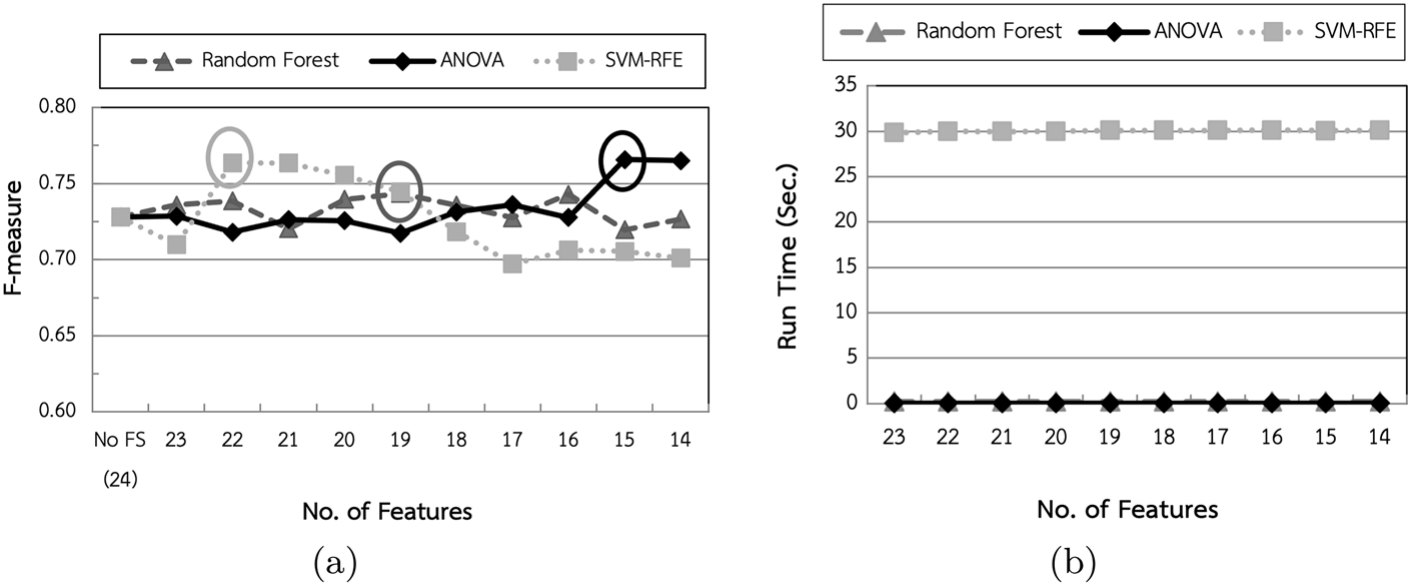
Feature selection performance: **a** F-measure; **b** processing time

In the second experiment, three feature selection methods, called Random Forest, Analysis of Variance (ANOVA) and Support Vector Machine-Recursive Feature Elimination (SVM-RFE), had been applied. The feature selection performance is shown in Fig. 6.

Figure 6a revealed that the optimal numbers of features for Random Forest, ANOVA and SVM-RFE were reduced from 24 features to 19, 15 and 22 features, respectively, while Fig. 6b revealed that feature selection using ANOVA was 7 and 917 times faster than Random Forest and SVM-RFE, respectively. The results also indicated that the number of features did not affect the processing time for feature selection. This experiment concluded that 19, 15 and 22 features were appropriate numbers for Random Forest, ANOVA and SVM-RFE methods, respectively. The results also reflected that the proposed model with feature selection using Random Forest (F-measure = 0.7435), ANOVA (F-measure = 0.7657), and SVM-RFE (F-measure = 0.7646) achieved better performance than existing models with no feature selection (F-measure = 0.728).

The third experiment measured model construction time and model usage time of Random Forest, ANOVA and SVM-RFE with the numbers of features based on the previous experiment.

**Fig. 7:**
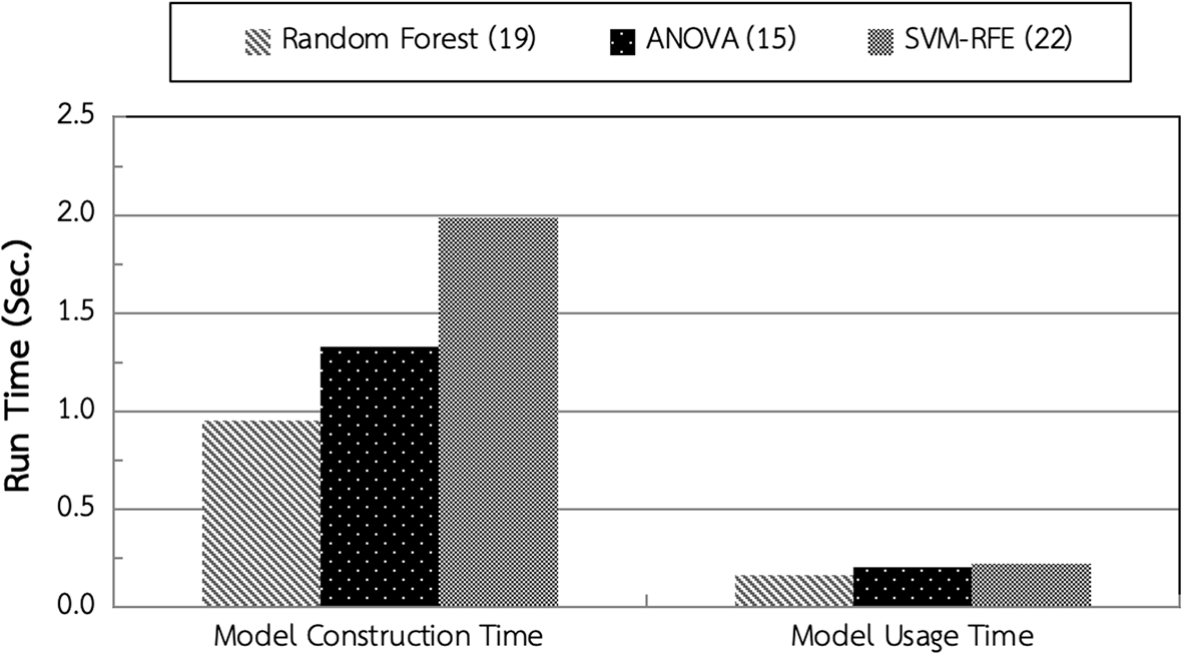
Model construction time and model usage time of feature selection

The experimental results as illustrated in Fig. 7 indicated that model construction and usage of ANOVA was faster than SVM-RFE, but marginally slower than Random Forest. However, ANOVA achieved the highest feature selection performance as shown in Fig 6. Hence, ANOVA with 15 features would be used as the feature selection process of this work.

**Fig. 8:**
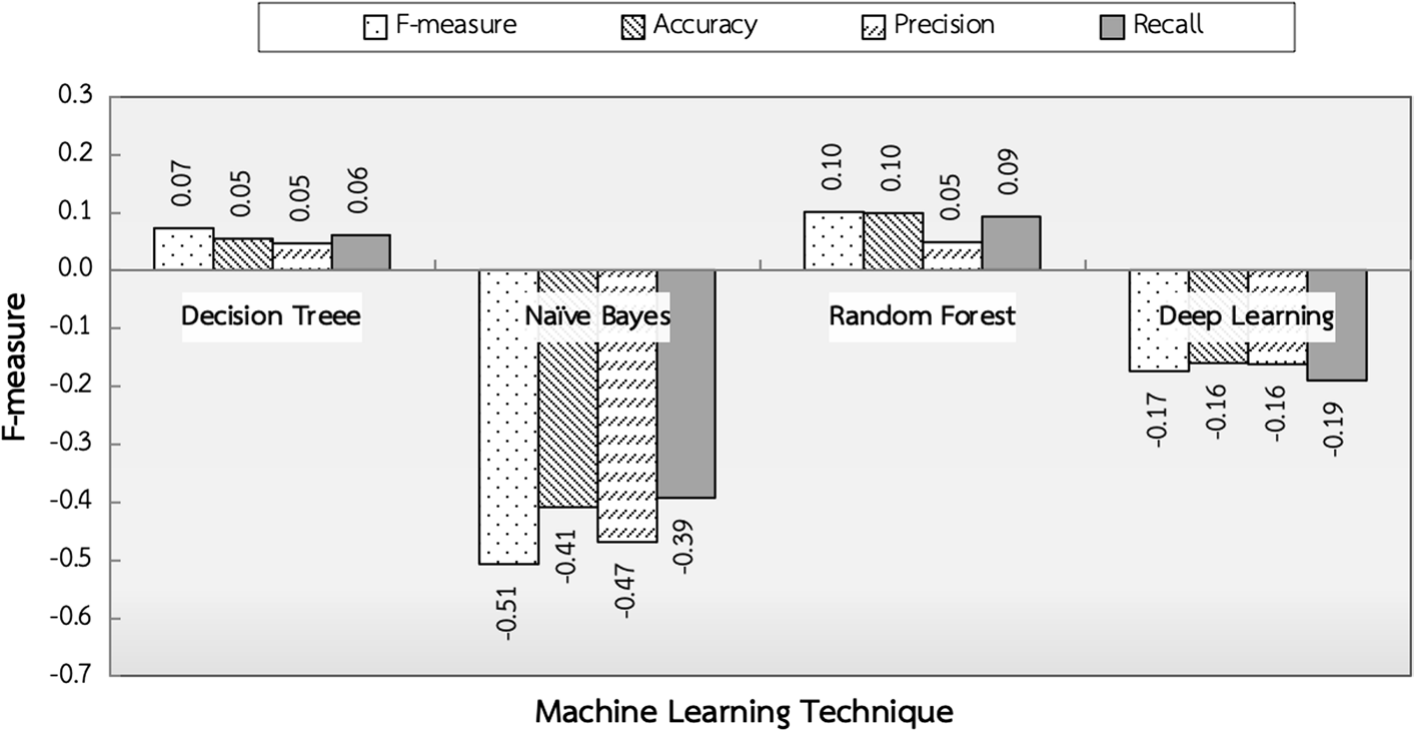
Performance comparison of machine learning techniques compared with Support Vector Machine

The last experiment aimed to find the most appropriate technique of machine learning for detecting the depression using standard measure scores which are f-measure, accuracy, precision, and recall. Fig. 8 showed that the performance of Decision Tree, Naïve Bayes, Random Forest, and Deep Learning techniques compared with Support Vector Machine, which was chosen as the baseline. The results revealed that Decision Tree and Random Forest techniques provided higher accuracy than the baseline. The Random Forest technique provided higher accuracy than the Decision Tree because it was an improvement from the decision tree technique. It was a simulation of multiple decision tree techniques. The advantage of this technique was that it provided accurate prediction results. Thus, the Random Forest was the most appropriate machine learning technique to detect the depression.

## Conclusions and future work

This article introduces a depression detection model on social networks using big data analytics. The main theoretical implication of this research is a novel model based on analyzing demographic characteristics and text sentiment of Twitter users. The two feature selection methods, called ANOVA and SVM-RFE, are applied to improve the performance of the model. The experimental results revealed that ANOVA achieved a higher f-measure, while consuming less processing time (including feature selection time, model construction time, and model usage time) than SVM-RFE. Then, machine learning techniques are applied for the depression detection model construction. There are five machine learning techniques explored in this research which are Support Vector Machine, Decision Tree, Naïve Bayes, Random Forest, and Deep Learning. The experimental results revealed that the Random Forest technique achieved higher accuracy than other machine learning techniques to detect the depression.

Nevertheless, the designed model has some limitations. This research uses information from Twitter users, who agree with the disclosure, to create a model. Additionally, the information used in this research in only one that is publicly available, e.g., opinion information (tweets, retweets, hashtags) and friends and follower information. The designed depression detection model cannot access information that users do not want to disclose or private information such as direct messages. It is possible that these messages will influence the analysis of depression.

There are some improvements that could be performed in the near future. Firstly, the number of participants could be increased for higher accuracy. Secondly, other features such as length of time spent on social networks and the number of likes on each post could be investigated for model construction. Thirdly, improving the model construction process using other data such as emoticons and images could be performed. Finally, data could be gathered from multiple social networks.

## Data Availability

Twitter data is not publicly available due to privacy reasons. Other data that support the findings of this study are available upon reasonable request.

## Abbreviations

ANOVA: Analysis of Variance
API: Application Programming Interface
HDFS: Hadoop Distributed File System
PHQ-9: Patient Health Questionnaire-9
SVM-RFE: Support Vector Machine-Recursive Feature Elimination
